# Responsive Boronic Acid-Decorated (Co)polymers: From Glucose Sensors to Autonomous Drug Delivery

**DOI:** 10.3390/s16101736

**Published:** 2016-10-19

**Authors:** Gertjan Vancoillie, Richard Hoogenboom

**Affiliations:** Supramolecular Chemistry Group, Department of Organic and Macromolecular Chemistry, Ghent University, Krijgslaan 281 S4, Ghent 9000, Belgium; gertjanvancoillie@hotmail.com

**Keywords:** boronic acid, glucose-responsive, self-assembly, polymers, drug delivery

## Abstract

Boronic acid-containing (co)polymers have fascinated researchers for decades, garnering attention for their unique responsiveness toward 1,2- and 1,3-diols, including saccharides and nucleotides. The applications of materials that exert this property are manifold including sensing, but also self-regulated drug delivery systems through responsive membranes or micelles. In this review, some of the main applications of boronic acid containing (co)polymers are discussed focusing on the role of the boronic acid group in the response mechanism. We hope that this summary, which highlights the importance and potential of boronic acid-decorated polymeric materials, will inspire further research within this interesting field of responsive polymers and polymeric materials.

## 1. Introduction

**Figure 1:**
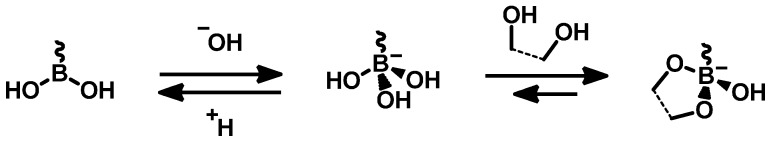
Simplified boronic acid equilibrium in the presence of a diol.

Boronic acid (BA) copolymers have been used for various applications over the years based on their responsiveness towards pH but also towards 1,2- and 1,3-diol concentration. The interaction of the boronic acid side chains with various mono-, di- and polysaccharides causes a shift in the boronic acid equilibrium through the formation of charged boronate esters. This shift increases the amount of negatively charged side chains and, therefore, the hydrophilicity of the entire (co)polymer (Figure 1) [1]. Depending on the architecture and structure of the copolymer in solution, this interaction can lead to swelling of cross-linked materials, but also to complete dissolution of polymeric aggregates. The synthesis and purification of these BA-functionalized (co)polymers are, however, notoriously difficult due to solubility issues and interactions of the Lewis acidic BA with the active center during polymerization. We have recently published a review covering the synthetic aspects of BA-containing polymers identifying three distinct synthetic pathways: (1) Direct polymerization of unprotected BA-containing monomers; (2) polymerization of protected boronic ester-containing monomers; and (3) post-polymerization modification reactions. While the first approach is the most straightforward, it is most commonly performed via free radical polymerization, although controlled radical polymerization under specific optimized conditions including high dilution and the presence of water to suppress crosslinking through boronic anhydride formation is also possible. The second approach reduces the influence of the BA moiety on the polymerization kinetics and increases its solubility in common organic solvents but also introduces an additional deprotection step to obtain the responsive copolymer. The third approach completely bypasses these problems by introducing the BA-moiety after polymerization through, e.g., amide-coupling reactions [2].

Although some applications like affinity chromatography or reactive surfaces focus on the specific interaction between BA moieties and a chosen diol, most applications are based on the pH- and saccharide-dependent polymeric phase transition in, e.g., responsive membranes, drug delivery and sensor applications [3,4,5,6]. In an attempt to highlight the possible applications, these three biomedically relevant applications will be discussed in this review. Because of the complex synthesis and corresponding response of BA-containing (co)polymers, multiple publications focus on the principles and control over the responsiveness while only suggesting possible applications. The papers that will be discussed in the first part of this review mostly aim at understanding the responsive polymeric phase transition characteristics of BA-containing (co)polymers and occasionally highlight their potential in, e.g., responsive surfaces [7,8], self-healable materials [9,10,11] or affinity chromatography. Specific examples of BA-containing (co)polymers in drug delivery and sensory applications will be discussed in the second and third part of this review, respectively.

## 2. Responsiveness of Boronic Acid Containing (Co)polymers

**Figure 2:**
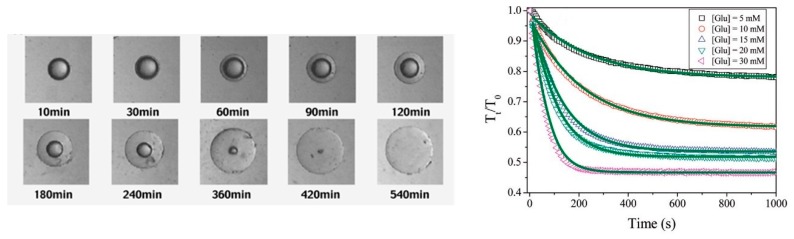
(**Left**) Images of swelling behavior of a P(NIPAAm-*co*-mAPBA) macrogel over time in the presence of 5 g/L d-Glu solution in pH 9 CHES buffer (T = 25 °C) [12]. Copyright 2004 American Chemical Society; (**Right**) Relative turbidity of P(NIPAAm*-co-m*APBA) microgel dispersion changes in function of time upon addition of d-Glu in 0.020 M pH 8.5 phosphate buffer (T = 25 °C) [13]. Copyright 2011 American Chemical Society.

The first commonly described reporter event in the field of BA-containing polymers is the swelling of a cross-linked polymer network. Such polymeric systems swell in the presence of glucose or the increase in pH due to the shift of the BA-equilibrium to the charged side, introducing a glucose-dependent permeability allowing its use in an autonomous insulin delivery device. The reversibility of such system would also diminish the release of the drug when the glucose is taken up by the cells and removed from the blood stream, preventing hypoglycemic shock. This possible application has fascinated researchers for years and sparked numerous publications related to the swelling behavior of cross-linked BA-containing polymer networks. Kataoka et al. were amongst the first to report the swelling behavior of a macrogel containing *N*-isopropylacrylamide (NIPAAm) and *meta*-acrylamidophenylboronic acid (*m*APBA) using microscope images in function of time (Figure 2, left). They showed that the used macrogel (D = 400 µm) took several hours to completely swell in the presence of a 5 g/L d-Glu concentration in a CHES buffered saline at an optimum pH of 9. The swelling rate was determined to be dependent on the size of the bead and the used d-Glu concentrations indicating that the penetration rate of d-Glu and the relaxation processes of the polymer chains during hydration play a vital role in the swelling kinetics [12]. Zhang et al. later focused on the swelling kinetics of P(NIPAAm*-co-m*APBA) microgels synthesized through free radical polymerization (FRP) in emulsion. The use of NIPAAm as comonomer allowed for the comparison of the thermal- and glucose-induced swelling kinetics of a dispersion of the microgel as measured using turbidimetry. They showed that complete swelling of the microgel took hundreds of seconds under the influence of glucose as shown in Figure 2 (right), which is 8–10 orders of magnitude slower than temperature induced swelling, identifying the rate-determining step to be the boronate ester formation as the delaying effect of the d-Glu penetration was limited due to the size of the microgel particles (D = 80 nm) [13]. Besides the obvious influencing parameters, the swelling speed shows a direct relationship with the pH of the aqueous solution originating from the BA equilibrium and an inverse relationship with the solution temperature caused by reduced accessibility of the BA groups in the temperature induced collapsed state [13]. This same material was used in the fabrication of glucose responsive microcapsules through oil/water/oil emulsion polymerization of NIPAAm, *m*APBA and a cross-linker in the water phase. The addition of glucose to these microcapsules results in the swelling of the membrane without losing its structural integrity allowing the release of the designed payload present in the inner cavity [14]. 

**Figure 3:**
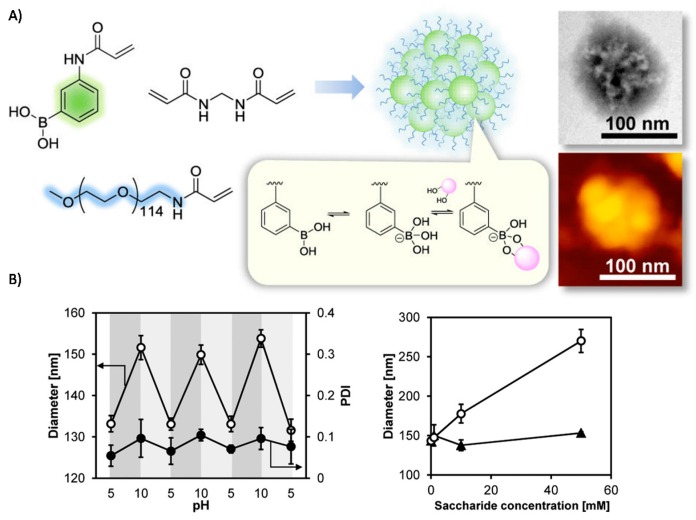
(**A**) Monomer structures and schematic representation of the polymeric framboidal aggregate with TEM (top) and AFM (bottom) picture; (**B**) Swelling behavior of the PBA-NPs with the reversible swelling behavior upon pH change between pH 5 and pH 10 as measured by DLS (left) and the diameter change of PBA-NPs upon the addition of d-glucose (triangle) and d-fructose (circle) as measured by DLS [16]. Copyright 2015 American Chemical Society.

To allow this swelling behavior under physiological condition (pH 7.4, 37 °C), Miyahara et al. fine-tuned the pKa of the used BA-monomer while retaining a similar swelling behavior of the developed 4-(1,6-dioxo-2,5-diaza-7-oxamyl)phenylboronic acid (DDOPBA)-containing polymeric gel as the previously discussed *m*APBA-containing macrogel [15]. A recent publication showed the formation of so-called framboidal nanoparticles (NP) using aqueous dispersion polymerization with a glucose-responsive *m*APBA rich core and water soluble methoxypolyethyleneglycol (mPEG) shell. The presence of the BA moieties allowed for the controlled and reversible swelling of the nanoparticles under the influence of pH and various saccharides. The results show reversible swelling of the PBA-NP with a particle diameter roughly alternating between 130 nm (pH 5) and 150 nm (pH 10). Similar swelling behavior was observed upon the addition of saccharides in aqueous solution (pH = 8.0) and subsequent incubation for 24 h at room temperature (rT) (Figure 3) [16]. These selected publications show the importance of the size of the synthesized material when considering the response time for the final applications. Most of the used copolymer gels are unfortunately synthesized using FRP, limiting the possible (co)polymer architectures and control over the copolymer size.

**Figure 4:**
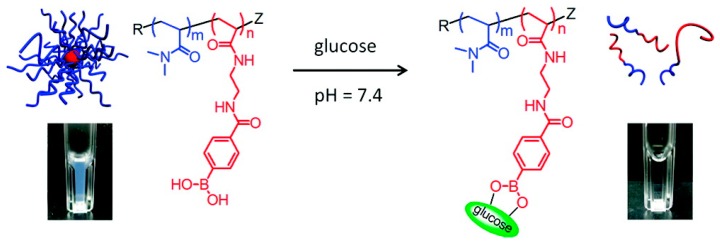
Structure and schematic representation of the micellar association/dissociation under the influence of glucose at physiological pH for P(DMA-block-DDOPBA) [21]. Copyright 2012 American Chemical Society.

The use of controlled radical polymerization (CRP) methods opened up a whole new range of polymeric architectures and reporter events. The pH-induced phase transition for non-cross-linked BA-containing homopolymers is macroscopic precipitation in aqueous solution where the critical pH is dependent on the presence and concentration of diols. This macroscopic precipitation can however be avoided by the incorporation of a fully water soluble polymer block like PEG or dimethylacrylamide (DMA). The use of such block copolymers allows the pH- and glucose dependent formation/dissociation of self-assembled aggregates like micelles and vesicles, which are commonly used in both sensor and drug delivery applications. One of the first to report responsive polymeric micelles from BA-containing block copolymers synthesized via CRP were Sumerlin et al. in 2007. Through reversible addition-fragmentation chain transfer (RAFT) polymerization of a pinacol protected 4-VBA that was subsequently chain extended with the water soluble DMA, an amphiphilic block copolymer was formed that self-assembled into micelles of about 100 nm diameter [17]. The reversible, glucose-induced dissociation was reported a year later for a P(DMA)-*block*-P(*m*APBA) showing uniform micellar like aggregates (D = 35 nm) at a pH of 8.7, which dissociate upon addition of 45 mM of d-Glu as measured by dynamic light scattering (DLS) [18]. The kinetics of the dissociation of these micelles were shown to be dependent on block copolymer structure, pH and the identity of the added sugar [19]. Similar amphiphilic block-copolymers were reported through the use of an mPEG-functionalized macro-chain transfer agent for the RAFT polymerization of 4-VBA [20]. As with the previously discussed application, the used *m*APBA is characterized by a pKa that prevents sufficient interaction with d-Glu under physiological conditions (pH 7.4) and requires slightly alkaline conditions (pH ≈ 8–8.5). The same micellar approach was made possible under physiological conditions by using a P(DMA)-*block*-P(DDOPBA) showing a strong decrease in particle size from 100 nm to unimers (6 nm) upon addition of glucose in an aqueous solution with pH 7.4 (Figure 4) [21]. Similar results were obtained for a block copolymer containing PEG and the glucose responsive 2-dimethylaminomethyl-5-vinylphenylboronic acid showing aggregation in PBS buffer (pH = 7.4, D = 335 nm) and dissociation into unimers upon addition of 10–20 mM of monosaccharides [22].

The use of CRP techniques also allowed the synthesis of more complex copolymers with, e.g., thermoresponsive properties. The design of the copolymer allows for the control of the thermoresponsive properties through pH and glucose concentration with the incorporation of BA moieties. A copolymer of NIPAAm/NIPMAAm with DDOPBA was demonstrated to show glucose controlled lower critical solution temperature (LCST) behavior under physiological conditions [23]. A more common combination of NIPAAm with *m*APBA showed remarkable control over the LCST transition of the formed copolymer using pH and d-Glu but also by variation of the concentration of RNA [24], d-fructose, ascorbic acid, nucleotides and other saccharide containing biomolecules including polyols and glycoproteins [25,26,27].

A final example of the use of boronic acid functional materials focuses on the specific interaction and retention of diol containing compounds. The interaction between the BA groups and various nucleotides allows the control over the swelling/deswelling as measured by the water content. These materials were used in affinity chromatography in order to quantitatively determine the content of the analyte or to selectively concentrate a desired diol-containing biomolecule [28,29]. Similar investigations were reported for a NIPAAm—4-vinylphenylboronic acid (4-VBA) gel for the interaction with RNA [30]. Most of the BA-functionalized materials are designed to retain or enrich a targeted, diol-containing biomolecule. For example, ordered mesoporous silica was functionalized with BA moieties through surface initiated atom transfer radical polymerization (ATRP) with the resulting highly porous material showing remarkable retrieval properties of d-Glu and d-Xyl in complex mixtures through boronate ester formation [31]. Similar functionalized materials were designed by Zheng et al. reporting the surface initiated ATRP of 4-VBA in order to create P(4-VBA) chain grafted poly(glycidylmethacrylate-*co*-ethylenedimethacrylate) beads. It was shown that these materials were suitable for the enrichment of glycoproteins in complex biological samples including human serum samples to allow straightforward glycoproteome analysis [32,33]. Similar results were obtained using BA-functionalized nanodiamonds allowing the recovery of up to 75% of glycopeptides in biological samples [34]. Due to the large amount of glycosylated peptides on the extracellular matrix of various cells, boronate ester formation can also be used to harvest cell sheets in a non-invasive way and detaching the sheet by addition of harmless saccharides [35].

## 3. Drug Delivery Applications

The use of responsive micelles for the delivery of hydrophobic drugs has been numerously reported in scientific literature, albeit there is some debate on the stability of such carriers in blood plasma [36]. In short, the drug can be loaded into the hydrophobic core of the micelles through physical interaction or chemical modification, increasing its solubility in aqueous environment and shielding it from enzymatic degradation. The drug is released when a specific stimulus triggers an increase in solubility of the responsive inner-block resulting in swelling or dissolution of the micelle core.

The most straightforward drug release mechanism is based on the immobilization of a suitable drug as boronate ester prior to use. An example of this mechanism was published by Sakurai et al. in 1994 employing a cross-linked hydrogel bead fabricated using suspension polymerization of *meta*-methacrylamidophenylboronic acid (*m*MAPBA), acrylamide and a cross-linker. Chemically modified insulin containing gluconic acid moieties (G-Ins) was subsequently immobilized as boronate ester overnight in buffer solution. The release of the G-Ins through competitive binding with free glucose in a HEPES buffered solution (pH 8.5) was monitored using fluorescence spectroscopy and showed a quick and d-Glu concentration dependent release profile [37,38]. This release mechanism was adapted to function under physiological conditions by incorporation of tertiary amine containing comonomers, which lowers the pKa of the boronic acid through interaction with the boron. The incorporation of the amine increased both the selectivity of the response showing better retention of the immobilized G-Ins in the absence of d-Glu as well as the sensitivity with increased release at lower d-Glu concentrations [39,40].

By careful selection of the used comonomer, further responsiveness can be added to the polymeric system influencing the drug release profile. An example of this has been reported by Sakurai et al. where they fabricated P(NIPAAm*-co-m*APBA) polymer gels introducing pH and glucose sensitivity on the LCST transition and vice versa. The added temperature control allows the on–off regulation of physically loaded fluorescently labeled insulin (FITC-Insulin) through precipitation of the gel, forming a barrier for further drug release. The addition of glucose increases the hydrophilicity of the copolymer gel through charged boronate ester formation and therefore increases the transition temperatures, solubilizing the gel at a fixed temperature [41]. Zhang et al. further explored upon this copolymer in microgels for the release of both chemically immobilized alizarin red S (ARS) and physically loaded FITC-insulin. By diluting the structure of the copolymer with acrylic acid, a more open structure of the precipitated polymer globules was obtained causing a squeeze-out release mechanism of both ARS and FITC-insulin upon temperature increase. The change in release profile upon addition of d-Glu was, however, strongly dependent on the type of immobilization since the added negative charges counteract the thermally induced “squeezing-out” of the drug. The chemically immobilized ARS showed an increase of release through competitive binding with the added saccharide while the physically loaded FITC-Insulin showed a decrease in release due to the swelling of the polymeric microgel caused by electrostatic repulsion [42].

**Figure 5:**
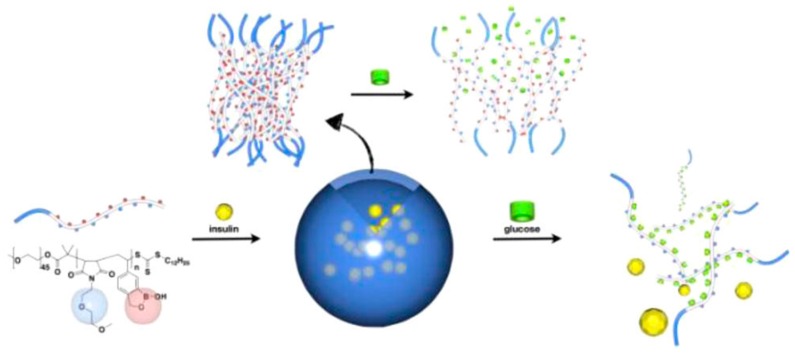
Schematic representation of glucose-triggered disassembly of polymersomes of sugar-responsive block copolymers [47]. Copyright 2012 American Chemical Society.

More complex polymer architectures prepared by CRP techniques allow for the use of more intricate self-assembled structures. An interesting example utilizes block copolymers containing a water-soluble PEG moiety and a responsive block constructed from phenylboronic ester side chains attached to the polymer backbone through the protective diol. The addition of d-Glu will release the phenyl boronic acid as a boronate ester, leaving behind a diol on the polymeric backbone, which increases the hydrophilicity of the inner micellar core [43,44,45]. Various other BA-containing block copolymers, for example including PDMA-*block*-P*m*APBA, showed the release of physically absorbed Nile Red in the micelle core in response to increasing pH or added glucose/fructose [19]. More complex release structures can be made by fine tuning the copolymer composition. This is highlighted by the work of Kim et al. on glucose-responsive block copolymers containing a polyboroxole (PBOx) as responsive part. mPEG_45_-*block*-PBOx self-assembled into various structures ranging from spherical and cylindrical micelles to polymersomes in aqueous conditions with increasing DP of BOx from 36 to 58. These structures could be loaded with ARS or FITC-insulin, which could subsequently be released upon the addition of fructose or glucose [46]. This research was further expanded towards the self-assembly of these block copolymers into polymersomes allowing for higher loadings. By performing an alternating copolymerization of styreneboroxoles with *N*-functionalized maleimides, various PEG containing block copolymers were synthesized that formed uniform polymersomes in both neutral buffer and serum (Figure 5). Due to the nature of the polymersomes, water-soluble drugs can be contained and shielded within the aqueous environment of the self-assembled core without the need for chemical modification [47]. 

**Figure 6:**
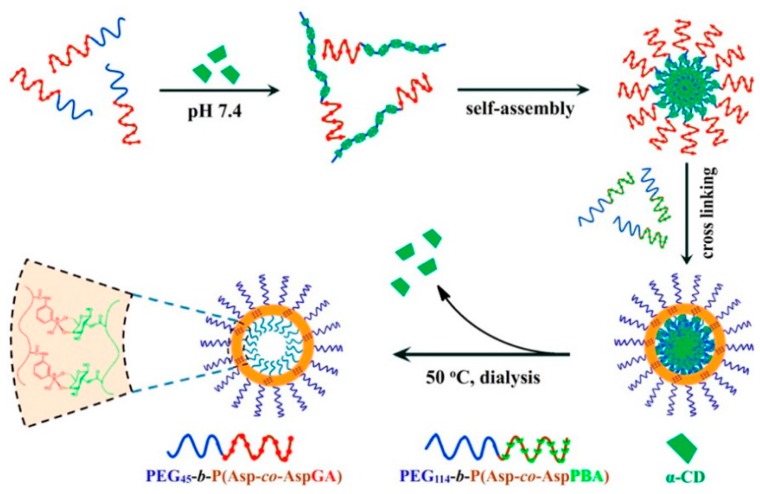
Schematic representation of the fabricated polymer vesicles using a αCD/PEG inclusion complex-template strategy [49]. Copyright 2015 American Chemical Society.

Besides the self-assembly of a single, responsive block copolymer, polymeric drug delivery systems can be created through interaction of two separate block copolymers. An example of this drug delivery strategy was reported by Shi et al. in 2013 where they showed the formation of polymeric micelles by combining two PEG-*block*-P(aspartic acid) based synthetic polypeptides modified with PBA or d-glucosamine (GA). The combination of both PBA and d-glucosamine functionalized copolymers in the correct ratio resulted in the formation of self-assembled micelles with a cross-linked core containing boronate ester linkages, which could subsequently be loaded with FITC-insulin. The addition of glucose resulted in the release of the loaded drug through competitive binding with the micelle cross-linking points under physiological conditions (pH 7.4) and with a biomedically relevant sensitivity towards the stimuli. They showed notable release of loaded insulin with a 2 mg/mL d-Glu concentration while the micelles were relatively stable around 1 mg/mL d-Glu [48]. A similar mechanism was used to fabricate uniform vesicles by first forming polymeric micelles with a PEG core and a glycosylated synthetic polypeptide (PEG-*block*-P(Asp-*co*-AspGA)) shell through cyclodextrin (CD) assisted self-assembly. The shell was subsequently cross-linked using a PEG-*block*-P(Aspartic acid-*co*-aspartamidophenylboronic acid) (PEG-*block*-P(Asp-*co*-AspPBA)) through boronate ester formation, after which the CD was removed using dialysis (Figure 6). The obtained self-assembled structure could be loaded with the glycopeptide antibiotic Vancomycin, which was slowly released over 14 h in the presence of saccharides like fructose and glucose through competitive binding [49].

The use of two interacting polymers for the fabrication of glucose responsive structures through competitive binding has been reported as early as 1991 by Sakurai et al. for the complexation between polyvinylalcohol (PVA) and P(*N*-vinylpyrrolidone-*m*APBA). By mixing the two polymers, a highly cross-linked gel was obtained that showed a strong decrease in viscosity upon addition of free d-Glu resulting from the competitive binding [50]. This approach has been adapted to be physiologically active by copolymerizing a tertiary amine into the BA containing copolymer yielding P(*N*-vinylpyrrolidone-mAPBA-DMAPAA). The formed gel could be loaded with myoglobin during the mixing of the BA-containing polymer with PVA in a 0.01 M phosphate buffer (pH 7.4), effectively trapping the protein drug inside the cross-linked network, which could subsequently be released upon addition of d-Glu [51,52]. This complex was further investigated by Mattiasson et al. using rheology and revealed the possibility of controlling the viscoelastic properties through pH and chemical composition [53]. These systems of interacting functional polymers are available in various forms using layer-by-layer assembly. Responsive hollow capsules were reported through the combination of a cationically charged dimethylaminoethylacrylate copolymer functionalized with *m*APBA and anionically charged polystyrene sulfonate. Upon addition of glucose, the capsule was shown to dissociate due to electrostatic repulsion and release its payload [54]. A similar layer-by-layer film was reported using PVA and a 4-carboxyphenylboronic acid-modified dendrimer revealing a strong concentration dependent, irreversible decomposition upon the addition of a saccharide [55]. The final example of a responsive application for a two polymer system has been described by Mattiason et al. where they use the gelation of a P(DMA-*co*-APBA) polymer with a PVA-borax gel to be used as a tissue sealant. The mixing of the polymers showed a remarkably quick gelation with little leakage of the occluded organs through formation of boronate esters with both the PVA polymer as well as the mucin in the epithelium of the organ ensuring a strong integration of the resulting gel plug. The occlusion could easily be removed by injection of an aqueous fructose solution [56].

Phenylboronic acid groups have also proven their usefulness in the field of gene transfection. Xu et al. recently showed that the introduction of BA groups into a four-arm star polycation improved the transfection efficiency through specific interaction with the glycoproteins on the cell-membrane [57]. This enhanced efficiency was also shown for a BA modified pluronic revealing strong interactions between the sugar-unit of the nucleotides and the incorporated 2-dimethylaminomethyl BA allowing the efficient condensation of DNA using a neutral polymeric vector under physiological conditions and in serum [58]. Similar results were obtained using polyethyleneimine as polymeric base [59].

**Figure 7:**
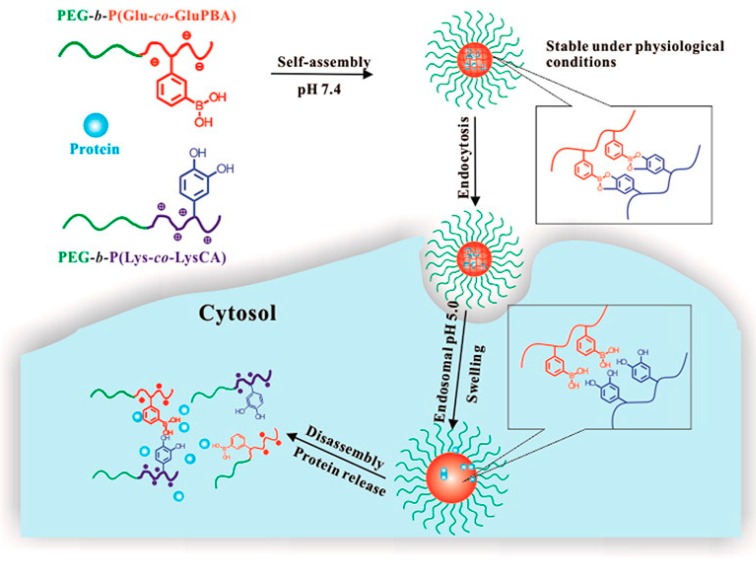
Schematic representation of a protein-loaded core-cross-linked micelle through boronate ester-catechol complex formation and subsequent intracellular protein delivery triggered by endosomal pH [61]. Copyright 2013 American Chemical Society.

The specific interaction of BA moieties with 1,2- and 1,3-diols was also employed for the delivery of siRNA duplexes through both electrostatic condensation with a peptide-based polycation and chemical modification of the ribose moieties at the 3’ end through boronate ester formation with BA moieties. The used PEG-*block*-P(Lys-*co*-fluoro-BA) is reversibly cross-linked by the added siRNA, protecting the payload in the extracellular matrix which is set free due to the competitive binding with other ribose containing molecules present inside the intracellular matrix. The stability of the formed boronate ester showed remarkable selectivity in the competitive binding with little release of siRNA upon addition of d-Glu while significant release occurred when adding other ribose containing biomolecules like ATP [60]. Another example of a peptide-based responsive polymeric assembly was published by Shi et al. combing poly(ethylene glycol)-*b*-poly(glutamic acid-*co*-glutamicamidophenylboronic acid) (PEG-*b*-P(Glu-*co*-GluPBA)) and poly-(ethylene glycol)-*b*-poly(llysine-*co*-ε-3,4-dihydroxyphenylcarboxyl-llysine) (PEG-*b*-P(Lys-*co*-LysCA)) copolymers. These copolymers assemble into core-cross-linked polyion complex micelles through the formation of boronate ester-catechol complexes. The formed assemblies could be loaded with FITC-labelled proteins, which could subsequently be released in vitro in response to endosomal acidic pH values or excess fructose through transesterification (Figure 7) [61]. Similarly, Zhuo et al. used PEG-*b*-P(Lys-*co*-LysCA) in combination with BA-modified cholesterol moieties to encapsulate poorly water-soluble drug like doxorubicin into a micellar structure and release it upon a decrease in pH [62]. 

Finally, BA functionalization has also been used as targeting agent for therapeutics. An example of this has recently been reported by Kataoka et al. using a polymeric micelle approach to deliver dichloro(1,2-diamino-cyclohexane)platinum(II) (DACHPt), which is the parent molecule of the anticancer therapeutic Oxaliplatin. Mixing this therapeutic with α-PBA-PEG-*block*-P(lglutamic acid) allows for the formation of micelles with a DACHPt core stabilized by electrostatic interactions and a PEG shell in aqueous environment. The incorporation of the BA moieties on the periphery of the micelles allows for specific interactions with sialic acid epitopes that are commonly overexpressed on tumor cells, resulting in effective targeted release of the drug [63].

## 4. Sensory Applications

Polymeric sensors that provide an unambiguous and quick visual feedback signal in response to the presence or concentration of a certain analyte are very powerful tools in the biomedical field. The reversible interaction between BA and sugars in general, provides a basis for the continuous monitoring of the concentration of these analytes using, e.g., colorimetric or fluorescent signals. The easiest method of fabricating a polymeric sensor for diols is to immobilize a molecular fluorescent sensor onto a polymeric backbone. This method was used by Taylor et al. to fabricate a selective sialic acid sensor using polyallylamine as base material. The used molecular sensor showed enhanced fluorescence of the anthracene moiety by blocking the photo induced electron transfer (PET) from the nearby tertiary amine through complexation with the formed boronate ester [64]. It was theorized that the nitrogen-boronate ester complexation ties up the electron lone pair, preventing PET and causing fluorescent enhancement of the anthracene moiety, but this mechanism has been drawn into question [65]. Another example of this approach employs a synthesized BA-functionalized azobenzene moiety immobilized on a polyethyleneimine backbone showing a pH- and glucose dependent UV-vis spectrum through the disruption of the complexation between the azo bond and the neutral boronic acid form [55]. The advantage of this method is that previously optimized fluorescent molecular sensors for saccharides can be used and further fine-tuned in terms of sensitivity and selectivity by introducing other functionalities in proximity to the BA, influencing its binding behavior and selectivity [64,66,67,68,69,70]. The main disadvantage of this method is that most common molecular sensors for saccharides are complex dyes requiring extensive synthesis and purification to be able to attach them to a polymer chain.

An easier and generally applicable method for generating a fluorescent sensor for saccharides is based on the competitive binding of the analyte with a fluorescent compound like alizarin red S (ARS). This catechol dye is known to show intramolecular quenching of the excited state (λ_ex_ = 460 nm) through a proton transfer from the phenol hydroxyl group to the ketone oxygen. Formation of a boronate ester with ARS then blocks this quenching, causing fluorescent enhancement on complexation with BA moieties [71]. This competitive binding strategy is frequently used to calculate the association constants of various boronic acid moieties towards a range of different potential binding agents through monitoring of the decrease in fluorescence [72,73,74,75,76]. The fluorescence quenching can also be employed to visually report the presence of saccharides through dissociation of the BA-ARS complex through competitive binding with the saccharide present [25,77]. 

**Figure 8:**
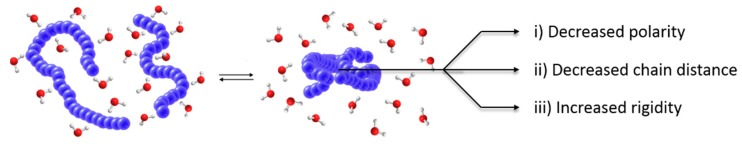
Schematic representation of a polymer phase transition in aqueous solution and a description of the significant changes in the local environment of the polymer. Adapted from [81].

Although there are several methods involving the modification of various surfaces with BA-moieties allowing the creation of saccharide sensors using gold electrodes [78], quartz crystal microbalance (QCM) [79] and organic field transistor [80], these mostly use the polymer as a structural supporting matrix and will not be reviewed here. However, the previously discussed glucose-induced polymeric phase transition can be translated into a colorimetric or fluorescent signal by incorporating suitable dyes in the polymer chain that target one of the following three characteristic changes (Figure 8): (1) upon precipitation, the inside of the collapsed polymeric globule strongly dehydrates leading to a decrease in polarity; (2) in a one-phase system, the polymer tries to maximize its contact with the solvent, taking on a very open and extended polymer structure, mostly represented as a random coil, and, as a result of the phase transition, this structure becomes increasingly dense, decreasing the inter- and intra-distance between the polymer backbone and its side-chains; and (3) this dense polymeric structure also strongly hinders the mobility of the side-chains, which is sometimes referred to as an increase in local viscosity, preventing, for example, free rotation between conjugated aromatic rings. Each of these concepts can be used as the basis of a colorimetric or fluorescent polymeric sensor by attaching a dye that responds to any of these changes to a responsive polymer. In this approach to polymeric sensors, the polymer not only has a structural role but also provides the main responsive mechanism allowing for the creation of sensors based on a wide variety of responsive polymers, most commonly temperature sensors, using this translation concept [81].

The first mechanism is perhaps the most complex and employs solvatochromic dyes to produce a colorimetric signal. Solvatochromism was first defined by Reichardt in 1994 as the change in fluorescence/absorbance of a molecule upon the change of the polarity of its microenvironment, attributed to the uneven stabilization of its ground state and first excited state. A larger stabilization of the ground state upon polarity increase will cause an increased energy gap between these two states, leading to a hypsochromic (blue) shift or so-called negative solvatochromism. The inverse situation in which the first excited state is stabilized more thus leads to a smaller energy gap and a bathochromic (red) shift or positive solvatochromism [81,82]. This translational mechanism has mostly been used for the creation of polymeric thermometers with examples including a 1,8-naphthalimide-decorated thermoresponsive copolymer like poly(*N*-isopropylacrylamide) showing fluorescent enhancement upon precipitation [83] or Disperse Red 1 (DR1)-oligoethyleneglycol acrylate copolymers showing a bathochromic shift [84,85]. The possibility of using a solvatochromic dye for the creation of polymeric sensors for glucose was published by our group in 2012 where the pH-induced polymer precipitation of a P(4-VBA) was translated into a color change by incorporating DR1 [86].

**Figure 9:**
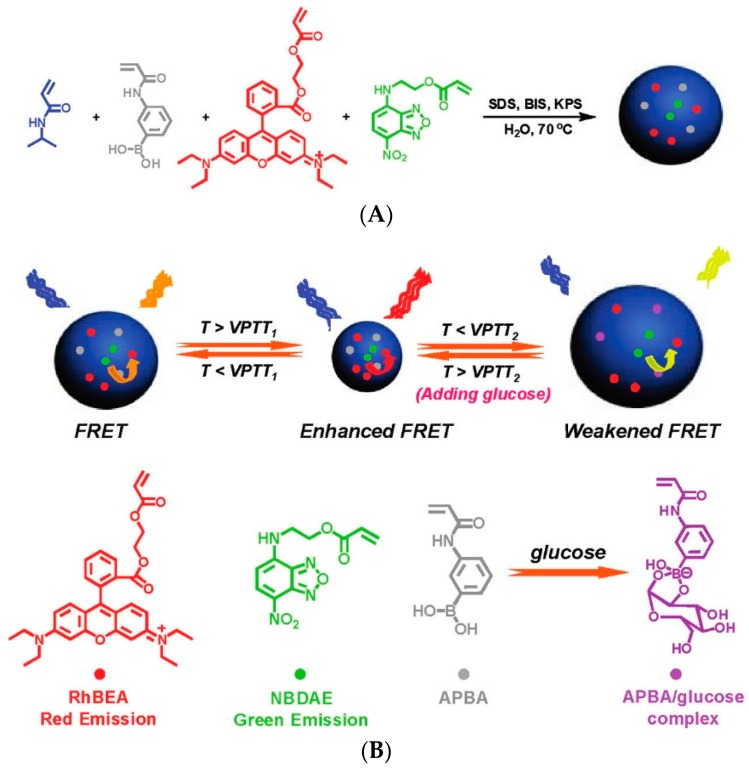
(**A**) Synthetic schemes employed for the preparation of thermo- and glucose-responsive P(NIPAM-APBA-NBDAE-RhBEA) fluorescent microgels via emulsion polymerization; (**B**) Schematic illustration for the modulation of FRET efficiencies within P(NIPAM-APBA-NBDAE-RhBEA) microgels by temperature variations and the addition of glucose [88]. Copyright 2011 American Chemical Society.

The second translation mechanism of sensing the decreased interchain distance can be achieved by a number of dyes or dye combinations. A first example uses Förster resonance energy transfer (FRET), which involves the non-radiative transfer of energy from an excited donor fluorophore to a second acceptor fluorophore that subsequently emits the transferred energy. This energy transfer is strongly affected by the distance between the two moieties and is only effective at very short distances [87]. This was employed by Liu et al. in their PNIPAAm-based microgel labelled with *m*PBA moieties and utilizing incorporated 4-(2-acryloyloxyethylamino)-7-nitro-2,1,3-benzoxadiazole (NBDAE, λ_ex_ 470 nm, λ_em_ 532 nm) as FRET donor and a Rhodamine B-derivative (RhBEA, λ_em_ 587 nm) as FRET acceptor (Figure 9). The emulsion polymerization employed in the synthesis of the microgel already places the FRET pair in close proximity providing a background energy transfer that however can be influenced by the volume change of the microgel. An increase in temperature to above the LCST of the copolymer causes the microgel to shrink, increasing the FRET and the fluorescence intensity of the RhBEA. The addition of glucose and the formation of charged boronate esters causes the swelling of the microgel due to electrostatic repulsion which can be monitored as a fluorescence quenching or decrease in the intensity ratio I_587_/I_532_ [88]. Another FRET pair involves the incorporation of the green dye 3,3′-dioctadecyloxacarbocyanine perchlorate (DiO) as donor in the core of the responsive micelle while RhB was introduced in an intermediate layer between the core and shell. Only when the environmental conditions allowed the aggregation of the block copolymer, is the RhB emission spectrum enhanced through FRET [89]. 

A second example of using the changing interchain distance to report the presence of glucose is based on the resonance energy transfer from a fluorescent dye to a quencher. An example of this has been reported by Singaram et al. where an intricately synthesized, viologen based quencher is modified with boronic acid moieties and subsequently introduced in a copolymer hydrogel with a negatively charged, pyrene-based fluorescent dye. In the absence of saccharides, the excitation of the dye is quenched by the viologen through electrostatic interaction. Upon addition of saccharides, the boronic acid moiety becomes negatively charged, disrupting the quenching of the dye leading to a fluorescent enhancement [90]. These thin film hydrogels were later applied to multi-well plates to allow high throughput analysis of solutions using common well plate readers [91].

**Figure 10:**
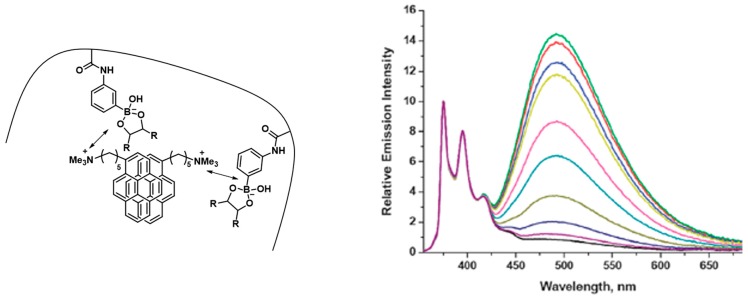
(**Left**) Schematic representation of electrostatically induced excimer formation of positively charged trimethylpentylammonium pyrene salt; (**Right**) Emission spectra (normalized at 375 nm) with increased glucose concentration showing excimer emission enhancement between 450 nm and 650 nm [93]. Reproduced by permission of The Royal Society of Chemistry.

A third example of a saccharide responsive polymeric system based on this mechanism uses so-called excimer formation/deformation of pendant pyrene moieties upon the binding of glucose. Thishas been reported by Tao et al. by copolymerizing a pyrene-acrylamide derivative with acrylamide and *m*APBA. By fine-tuning the identities of the comonomer, both excimer formation and deformation upon saccharide interaction can be promoted. In the first case where the polymer backbone bears no charge in the absence of saccharide, the polymer gel would collapse causing the pyrene moieties to form excimers. Upon addition of glucose, the formed negative charges would repulse the copolymer chain and allow the material to swell, breaking apart the excimers. The reverse system is possible when a cationically charge comonomer is incorporated into the responsive gel, showing the spectrum of isolated pyrene moieties in the absence of saccharides. The electrostatic interaction with the formed charged boronate esters upon diol addition promotes the formation of pyrene excimers, which showed the most sensitive result with charged diols like calcium-glucarate [92]. This final system where the boronate ester formation promotes excimer formation has also been reported by Yam et al. based on a positively charged trimethylpentylammonium pyrene salt that is forced to interact through electrostatic interactions with the negatively charged boronate esters (Figure 10, left). Upon addition of glucose, the emission spectrum (λ_ex_ = 350 nm) of the aqueous polymer solution shows an increased emission intensity of the characteristic broad excimer band between 450 nm and 650 nm (Figure 10, right) [93].

**Figure 11:**
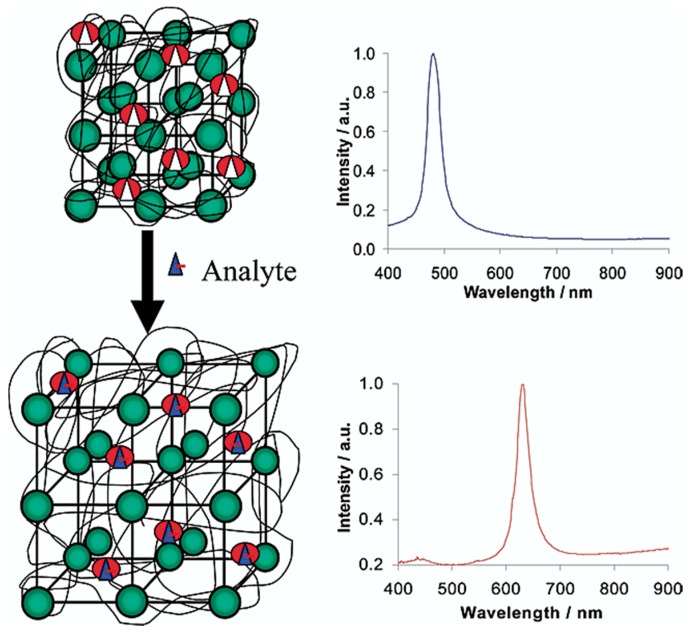
(**Left**) PCCA photonic crystal sensing materials consist of an embedded CCA surrounded by a polymer hydrogel network that contains boronic acid moieties (red circles). The imbedded PS-particles (green circles) diffract light of a wavelength determined by the array lattice constant; (**Right**) The change in diffracted wavelength resulting from the hydrogel volume swelling upon interaction with glucose as analyte [94]. Copyright 2003 American Chemical Society.

A final saccharide sensor that will be discussed translates the volume change of a BA functionalized hydrogel upon interaction with various diols. The general mechanisms is based on the swelling and deswelling of a polymerized crystalline colloidal array (PCCA). For this, a crystalline colloidal array of polystyrene particles was embedded in a responsive polymeric hydrogel through free radical polymerization. The incorporation of boronic acid moieties in the hydrogel causes swelling of the PCCA upon addition/binding of saccharide. This alters the crystalline colloidal array (CCA) spacing, changing the lattice constant and generating a red shift in the visible output signal of the sensor caused by the Bragg diffraction. Since the boronate ester formation between the BA moiety and the saccharide is completely reversible, removal of the analyte will cause a blue shift due to shrinking (Figure 11) [94,95]. Further optimization of the pKa of the boronic acid allowed for a PCCA led to large diffraction shifts (Δλ = 150 nm) as a result of 10 mM d-Glu under physiological conditions [96] and improved the response rate of the polymeric sensor [97]. A similar sensor was published by Kataoka et al. in 2003 showing a visibly noticeable color shift from green to red upon a glucose concentration increase from 5 to 20 mM in buffer solution by combining silica-based colloidal crystals imbedded in a 3-acrylamidophenylboronic acid-containing hydrogel [98].

The same principle of changing lattice constants was used by Lowe et al. for the creation of so-called glucose-sensitive holographic sensors. For this, a copolymer thin film consisting of cross-linked acrylamide and *m*APBA was imbedded with a silver nanoparticle lattice [99]. This has also been done with 4-VBA [100], 5-F-2MAPB [101] and a quaternary amine-*m*APBA copolymer for enhanced selectivity towards glucose [102,103]. The same group expanded on the BA monomers for these holographic applications by comparing *m*APBA and *o*APBA copolymers. It was reported that while the *m*APBA copolymer has a pH-dependent glucose complexation, the *o*APBA show a constant binding affinity in function of pH as a result of the intermolecular coordination between the carbonyl oxygen and the boron [104].

The third and final mechanism to translate the polymer phase transition in an output signal is based on the decreased mobility of the polymer chains and pendant side groups within the precipitated polymeric globule. An example has been published by Tang et al. using tetraphenylethene (TPE) dyes, a so-called aggregation induced emission (AIE) dye [105], which are known to dissipate their energy upon excitation through non-emissive rotation of the phenyl rings and an LCST copolymer [105]. Upon precipitation of the TPE-functionalized PNIPAAm, the increased rigidity restricts the free rotation of the phenyl rings around the central double bond causing a fluorescent increase [106,107]. The possibility of using such change in mobility induced response mechanism for polymeric glucose sensors was presented by our group in 2012 for a P(4-VBA) copolymer decorated with the home-made, fluorescent 4-(4-hexyl-5-(4-vinyl-phenyl)thiophen-2-yl)-7-(4-hexylthiophen-2-yl)-benzo[c]-[1,2,5]-thiadiazole (TBTS) [86]. Similarly to other dithiophene structures [108], fluorescent quenching was observed upon precipitation of the responsive copolymer.

## 5. Conclusions

This review focuses on various glucose-responsive applications of BA-containing (co)polymers, highlighting the importance and potential of BA-chemistry in the biomedical field. The reported swelling/deswelling or solubilization/precipitation of polymeric materials in the presence/absence of glucose not only provides a basis for autonomous drug delivery system but also for various types of sensing through the incorporation of suitable translator dyes. By carefully selecting a dye that shows a colorimetric/fluorescent response towards either the changing polarity, interchain distance or rigidity of the polymeric microenvironment upon the polymeric phase transition, the concentration of glucose or saccharides in general can easily be monitored using a visual or fluorescent reporter signal. The current challenges in this field of boronic acid based responsive materials for biomedical applications are still daunting. The logical next step of the discussed copolymers is to move towards in vivo-compatible and applicable sensing materials. In our opinion, the biggest limitation to current research is the use of strongly controlled testing environments to show proof-of-concept sugar sensing, which commonly consists of a buffered-solution with discrete concentrations of a single saccharide. The biggest challenge for in vivo applications lies in the investigation of the potential interference and competitive effects of all present biomolecules that can interact with BA-containing polymers and the fine tuning of the BA-materials to tailor the response to one single component, such as glucose.
